# Timed “ Up and Go” test in single and dual‐task conditions in older people with and without cognitive impairment according to Verbal Fluency Screening

**DOI:** 10.1002/alz.084147

**Published:** 2025-01-03

**Authors:** Beatriz S. E. Baccani, Sheila de Melo Borges

**Affiliations:** ^1^ Santa Cecilia University, Santos, Sao Paulo Brazil; ^2^ Santa Cecilia University, Santos Brazil

## Abstract

**Background:**

Motor performance is related to executive function, especially in dual tasks conditions. Thus, the aim of this study was to compare the results of Timed “Up and Go” test with and without dual task in older people with and without cognitive impairment according to verbal fluency test.

**Method:**

A cross‐sectional study was carried out in 291 elderly people attended by the Basic Health Unit of the city of Santos, São Paulo, Brazil. These elderly patients were divided in two groups, according to the presence of cognitive impairment (SG; n = 106) and the group without cognitive impairment (CG; n = 185) using Verbal Fluency (VF) test – animals category. The Timed “up and go” (TUG) test was evaluated under three experimental conditions: TUG‐simple (TUG alone); TUG‐cognitive (TUG with animal fluency task); and, TUG‐manual (TUG while carrying a full cup of water). The Mann‐Whitney test was used to compare TUG test between the groups (SG and CG) and ANCOVA for adjustment of the variable (age). The numerical data was presented with mean [minimum‐maximum] between groups.

**Result:**

The results showed a significant difference between groups on the development of TUG simple (SG = 11.17[6.63‐33.26] seconds, and CG = 9.79[5.0‐17.25] seconds ‐ p<0.001), TUG‐cognitive (SG = 13.29[6.07‐60.02] seconds, and CG = 11.76[4.15‐28.88] seconds ‐ p<0.001); and, TUG‐manual (SG = 12.29[7,13‐38,70] seconds, and CG = 10.5[4.75‐19.85] seconds ‐p<0.001) and this values remained significant after ANCOVA for adjustment of age (p<0.001).

**Conclusion:**

Older adults with cognitive impairment evaluated by VF test presented worse motor performance in both, with and without dual‐task conditions as compared to older person without cognitive impairment.

